# Co-expression of PD-L1 and HIF-1α predicts poor prognosis in Patients with Non-small Cell Lung Cancer after surgery

**DOI:** 10.7150/jca.53119

**Published:** 2021-02-02

**Authors:** Hongmei Zheng, Yue Ning, Yuting Zhan, Sile Liu, Yang Yang, Qiuyuan Wen, Songqing Fan

**Affiliations:** Department of Pathology, The Second Xiangya Hospital, Central South University, Changsha, Hunan, 410011, China.

**Keywords:** PD-L1, HIF-1α, biomarkers, Non-small cell lung cancer.

## Abstract

**Purpose:** PD-L1 is highly expressed in multiple cancers and suppresses anticancer immunity. HIF-1α, as a vital transcription factor, could regulate the proliferation, metastasis, and apoptosis of cancer cells. The aim of this study was to explore the correlation between PD-L1 and HIF-1α protein and further estimate its clinicopathological/prognostic impact on NSCLC patients. **Methods:** In this study, expression of PD-L1 and HIF-1α protein was detected by immunohistochemistry in tissue microarrays of NSCLC and non-cancerous tissues. **Results:** Expression of PD-L1 and HIF-1α protein was evidently elevated in NSCLC tissues compared with non-cancerous control lung tissues (both *P*<0.05). Also, PD-L1 was higher in male, lung SCC patients with lymph node metastasis (all *P*<0.05). There was a positive link between PD-L1 and HIF-1α in NSCLC (r=0.177, *P*=0.005). What's more, overall survival of lung ADC patients had to do with PD-L1 and clinical stage, while that of SCC patients was related to HIF-1α, pathological grade and LNM status (all *P*<0.05). Furthermore, multivariate analysis confirmed that PD-L1 and HIF-1α were considered to be independent prognostic factors for NSCLC patients (both *P*<0.05). **Conclusion:** PD-L1 and HIF-1α may serve as attractive independent worse prognostic biomarkers for NSCLC patients and the combined evaluation of PD-L1 and HIF-1α may also be valuable for prognosis judgment. Additionally, expression of PD-L1 was positively correlated with HIF-1α, which may provide evidences for a novel combinational therapy targeting PD-L1 and HIF-1α in NSCLC patients.

## 1. Introduction

Lung cancer is the main causes of cancer-related death worldwide and the incidence is increasing. Lung cancer is generally divided into SCLC (small cell lung cancer) and NSCLC (non-small cell lung cancer) in view of histological characteristics. NSCLC is usually classified into adenocarcinoma (ADC), squamous cell carcinoma (SCC) and large cell carcinoma 1. NSCLC accounts for about 80% of lung cancer, whose treatment has been a worldwide problem. Most patients are in advanced stage and have distant metastasis at the first diagnosis, resulting in a low 5-year overall survival rate 1, 2. Tyrosinase inhibitors (TKIs) targeting EGFR (epidermal growth factor receptor) have become a major breakthrough in the treatment of NSCLC. However, drug resistance has become a new problem with the widespread clinical application 3. Thus, finding some new biomarkers for predicting the occurrence and development and new therapeutic targets of NSCLC might be beneficial in improving the survival rate.

Programmed death-ligand 1 (PD-L1, also known as CD274 or B7-H1), as the major ligand of PD-1, is usually expressed on antigen-presenting cells as well as on tumor cells, which can bind to PD-1 to induce T-cell apoptosis and exhaustion, thereby suppressing anticancer immunity 4, 5. Also, tumor-intrinsic PD-L1 signaling plays a vital role in promoting occurrence, development and resistance to therapy by increasing MDR1 expression and activating of MAPK/ERK and PI3K/AKT pathways 6. Although immunotherapy targeting PD-1/PD-L1 axis has achieved great clinical success, a large proportion of patients, even those who express PD-L1^+^/PD-1^+^, have no respond to PD-1/PD-L1 blockade 7. Therefore, it is urgent to clarify the molecular mechanism and clinical characteristics of PD-L1, and to search for new biomarkers to jointly predict the efficacy of immunotherapy and the prognosis of NSCLC patients. HIF-1α (Hypoxia-inducible factor-1α) is a key nuclear transcription factor mediating hypoxic response of cancer cells, which can promote metabolism, angiogenesis and proliferation of cancer cells in hypoxic environment 8. More and more studies have shown that high HIF-1α expression is related to poor outcomes of many cancers, including lung cancer 9, 10. It has also been reported that HIF-1α can regulate the expression of PD-L1 at transcriptional level, and thereby increase the tolerance of cancer cells to cytotoxic T lymphocytes (CTL)-mediated lysis and drive immune escape 11.

However, to date, the correlation between PD-L1 and HIF-1α protein in NSCLC has not been well-investigated. Also, the relationship between these two proteins and clinicopathological features/prognosis of NSCLC patients has not been reported. Therefore, in this study, we detected the expression of PD-L1 and HIF-1α protein by IHC in TMAs (tissue microarrays) of NSCLC and explored their potential prognostic value in NSCLC patients, including lung SCC and ADC patients.

## 2. Materials and methods

### 2.1. Patient data and tissue microarrays (TMAs)

We collected 256 NSCLC patients who underwent surgery and 103 cases of non-cancerous control lung specimens at The Second Xiangya Hospital of Central South University (Changsha, China) between 2002 and 2012. All tumors were evaluated by experienced pathologists according to the WHO histological classification of the lung cancer and tumor stage was determined based on the Eighth Edition Lung Cancer 12. No patients had previously been treated with radiotherapy and/or chemotherapy at the time of original operation and none of them had been treated with agents targeting PD-1/PD-L1 during the follow-up time. Overall survival time was defined as the time from diagnosis to death or the last known moment of survival. This study was approved by the Ethics Committee of The Second Xiangya Hospital of Central South University (No: S039/2011) and complete clinical and follow-up data were available for all patients with written informed consent. The patient demographics were as follows: 191 males and 65 females, with an average age of (56.0 ± 8.8) years, 115 cases of patients with clinical stage III and 141 cases with stage I and II, 133 cases of ADC and 123 SCC. At the end of the follow-up, 172 patients survived and 84 died. All dead patients died of lung cancer. Tissue microarrays were made according to the technology as previously described 13.

### 2.2. Immunohistochemistry and scores

The IHC staining for samples on the TMAs was carried out with the ready-to-use Envision TM^+^ Dual Link Systenm-HRP methods (Dako, Carpinteria, CA). As described in detail previously 14. A 1:100 dilution of the primary antibody to PD-L1 (Rabbit monoclonal antibody, Catalogue Ab228462; Abcam, Cambridge, UK) and a 1:200 dilution of the primary antibody to HIF-1α (Rabbit monoclonal antibody, Catalogue Ab51608; Abcam, Cambridge, UK) were applied to test the expression of these two proteins. Positive control slides were included in every experiment. The specificity of the antibody was determined with matched IgG isotype antibody as negative control.

Expression of PD-L1 and HIF-1α was evaluated independently by SF and HZ, who were blinded to the clinicopathological data, at 200x magnification light microscopy. The evaluation method was as follows: Staining intensity for HIF-1α and PD-L1 of tumor cell was negative (-), weak (1+), moderate (2+) and strong (3+) 5, 14. For PD-L1 staining, cell surface membrane staining > 5% was considered as positive, while positive expression of HIF-1α was defined as IHC 1+, 2+ or 3+ regardless of the percentage of positive-stained cells. PD-L1 and HIF-1α was divided into negative expression and positive expression. Agreement between the two evaluators was 95%, and all discrepancies were resolved through discussion.

### 2.3. Statistical analysis

All statistical analyses were performed using SPSS 24.0. The chi-square test was used to explore the association between clinicopathological features and PD-L1/HIF-1α expression. The Spearman's rank correlation coefficient was used to estimate the correlation between expression of PD-L1 and HIF-1α. Kaplan-Meier analysis was performed for overall survival curves, and statistical significance was assessed using the log-rank test. Cox comparative hazards model was evaluated for multivariate analysis of independent prognostic factors. A two-sided p-value of less than 0.05 was considered statistically significant.

## 3. Results

### 3.1. Relationship between expression of HIF-1α and PD-L1 protein and clinicopathological features in NSCLC

**Figure 1:**
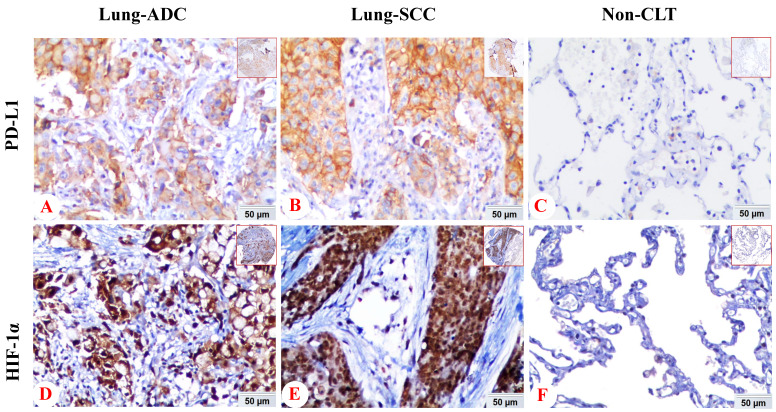
** Expression of PD-L1 and HIF-1α protein in lung ADC, lung SCC and Non-CLT (non-cancerous lung tissues) was detected by IHC.** Positive expression of PD-L1 (A) and HIF-1α (D) protein was shown in lung ADC. And positive expression of PD-L1 (B) and HIF-1α (E) protein was shown in lung SCC. Negative control staining of PD-L1 (C) and HIF-1α (F) protein was found in non-cancerous lung tissues. (IHC, DAB staining, original magnification ×200 and ×40).

In NSCLC tissues, positive expression of PD-L1 protein was discovered in the cell membrane/cytoplasm of tumor cells, while the HIF-1α protein was basically identified in the cell nucleus, also found in cytoplasm. There was no positive staining of IgG isotype antibody as negative control in the NSCLC and non-cancerous lung tissues (Figure 1).

**Figure 2:**
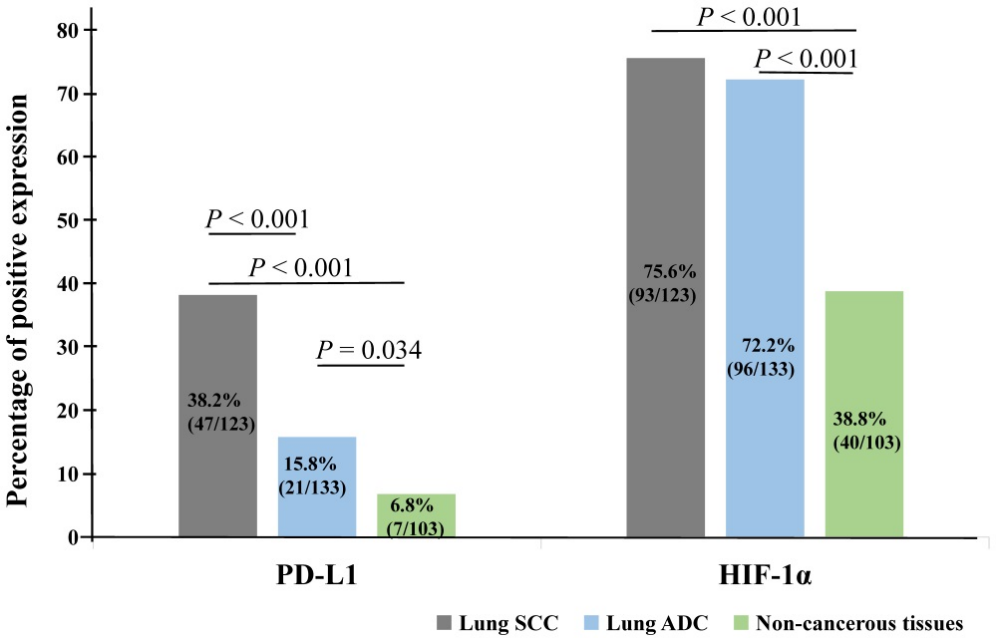
** The comparison of expression of PD-L1 and HIF-1α protein in lung SCC and lung ADC compared with non-cancerous lung tissues.** The expression of PD-L1 and HIF-1α protein in lung SCC and lung ADC was significantly higher than those in non-cancerous lung tissues (all *P* < 0.05). Moreover, the expression of PD-L1 was higher in lung SCC than lung ADC, which was statistically significant (*P <* 0.001).

We also analyzed the expression of HIF-1α and PD-L1 protein in lung SCC, ADC and non-cancerous lung control tissues. PD-L1 protein exhibited positive expression rates in non-cancerous lung tissues, lung SCC and ADC with values of 6.8% (7/103), 38.2% (47/123), 15.8% (21/133), respectively. While the positive expression rate of HIF-1α protein was 38.8% (40/103), 75.6% (93/123) and 72.2% (96/133) in non-cancerous lung tissues, lung SCC and ADC. Expression of PD-L1 and HIF-1α protein was evidently higher in lung ADC and SCC tissues (*P* < 0.05), which was shown in Figure 2.

We further explored the association between clinicopathological features and the expression of PD-L1 and HIF-1α protein, which mainly contained histological type, pathological grade, lymph node metastasis (LNM) status, age, gender and clinical stage (Table 1). Our results showed NSCLC patients with LNM (32.1%, 45/140) had significantly increased expression of PD-L1 protein than patients without LNM (19.8%, 23/116) (*P* = 0.026). And patients with lung SCC (38.2%, 47/123) had higher expression of PD-L1 than patients with lung ADC (15.8%, 21/133) (*P <* 0.001), while female patients (15.4%, 10/65) had lower expression than male patients (30.4%, 58/191) (*P =* 0.018). The expression of PD-L1 protein had no significant difference with age, clinical stage and pathological grade (all* P*>0.05). Also, no significant differences were seen between HIF-1α protein and clinicopathological features of NSCLC (all *P*>0.05). Co-expression of PD-L1 and HIF-1α protein was expressed in 59 of 256 samples (23.0%) with significant correlation with LNM status (LNM vs. No LNM: 28.6% (40/140) vs. 16.4% (19/116); *P =* 0.021), histological type (ADC vs. SCC: 13.5% (18/133) vs. 33.3% (41/123); *P <* 0.001) and gender (Female vs. Male: 13.8% (9/65) vs. 26.2% (50/191); *P =* 0.041).

### 3.2. The correlation between HIF-1α and PD-L1 protein in NSCLC

The correlation between PD-L1 and HIF-1α protein in NSCLC, consisting of lung ADC and lung SCC, was shown in Table 2. Data indicated that there was a significant positive correlation between PD-L1 and HIF-1α protein in NSCLC (r = 0.177, *P* = 0.005), which was also observed in lung SCC (r = 0.218, *P* = 0.003). However, no significant link was seen in lung ADC (r = 0.131, *P* = 0.134).

### 3.3. Impact of PD-L1 and HIF-1α protein on the prognosis of NSCLC patients

**Figure 3:**
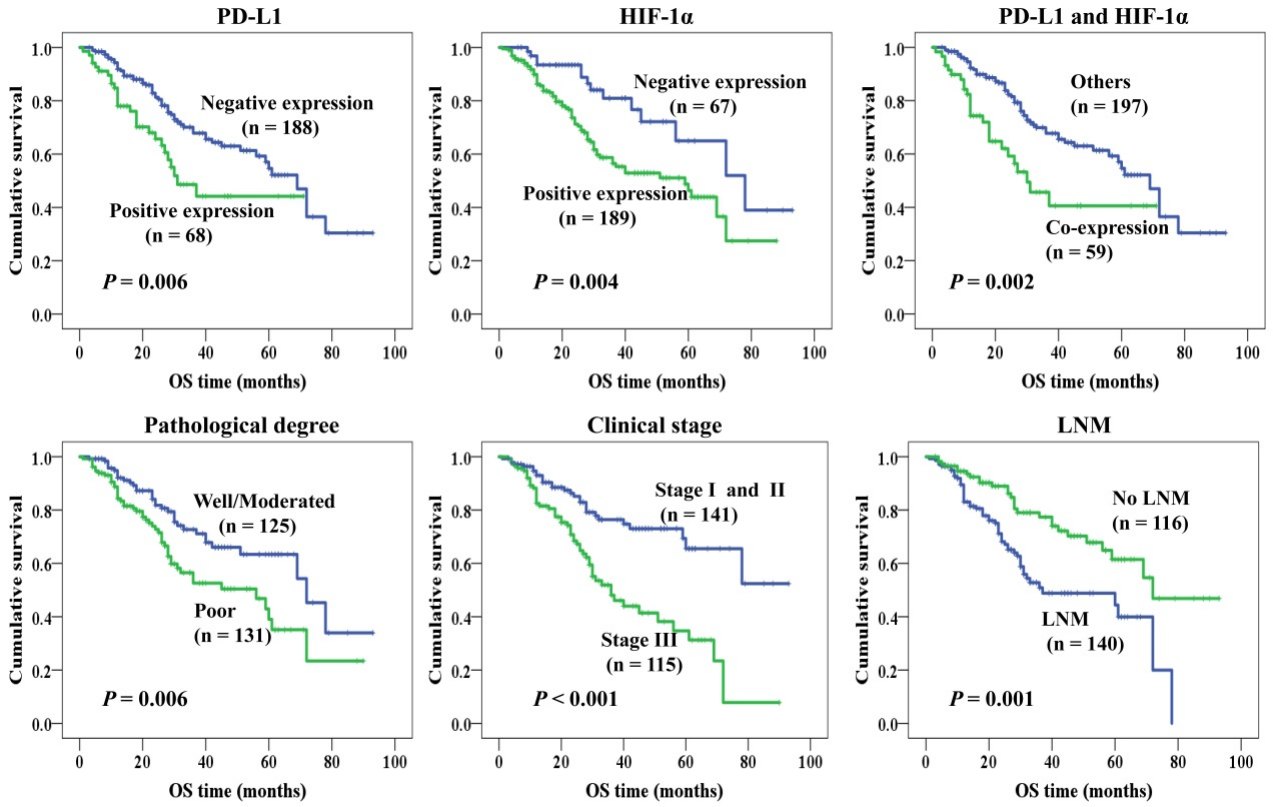
** Kaplan-Meier curves for overall survival of NSCLC patients.** Patients with positive expression of PD-L1 (A, *P* = 0.006), HIF-1α (B, *P* = 0.004) and co-expression of PD-L1 and HIF-1α (C, *P* = 0.002) had shorter survival time, as well as poor differentiation (D, *P* = 0.006), stage III (E, *P <* 0.001) and with LNM (F, *P* = 0.001).

Kaplan-Meier analysis was applied to examine the impact of PD-L1 and HIF-1α protein on the overall survival (OS) rate of NSCLC patients and using the log-rank test to assess statistical significance. Our results showed that patients with PD-L1 (Figure 3A) and HIF-1α (Figure 3B) protein expression had shorter survival (*P* = 0.006, *P* = 0.004, respectively). Also, patients with co-expression of HIF-1α and PD-L1 had shorter survival (*P* = 0.002, Figure 3C). However, higher OS rate could be seen for patients with well and moderated differentiation (*P* = 0.006, Figure 3D). Moreover, we also observed that patients with stage I and II had higher OS rate and the OS rate was longer for patients without LNM (*P* < 0.001, Figure 3E; *P* = 0.001, Figure 3F, respectively). But there were no significant impact on the histological type, age and gender (all *P >* 0.05).

To further confirm the prognostic effects of PD-L1 and HIF-1α protein, Cox proportional hazard regression was used for multivariate analysis, as shown in Table 3. We found positive PD-L1 and HIF-1α protein expression were identified as worse prognostic factors (*P* = 0.024, *P* = 0.010), as well as stage III (*P* = 0.003) and poor differentiation (*P* = 0.019). LNM tended to be a poor prognostic factor, but there was no statistical difference (*P* = 0.053). No clinical impact was observed in age, gender and histological type (all *P* > 0.05). Additionally, we have further analyzed the prognostic value of PD-L1 and HIF-1α protein in lung SCC and ADC respectively, which was shown in Supplementary Table S1. We found that overall survival of ADC patients was significantly associated with PD-L1 and clinical stage, while that of SCC patients was related to HIF-1α, pathological grade and LNM status (all *P* < 0.05).

## 4. Discussion

Lung cancer is one of the malignant tumors characterized by an aggressive clinical course and poor survival. PD-L1 can bind to PD-1 (programmed death-1) to induce T-cell apoptosis and exhaustion, thus promoting the immune escape of the tumor. In addition, tumor-intrinsic PD-L1 pathway is inappropriately activated and clearly contributes to epithelial-mesenchymal transition (EMT), cancer stemness, invasion and chemoresistance in multiple types of tumors 6. In the recent years, agents that target the PD-L1/PD-1 axis were revealed to improve the survival of solid cancer, including NSCLC 15, 16. Therefore, to further understand the function of PD-L1 will contribute to better immunotherapy and improve the safety of treatment. It was reported that PD-L1 protein was chiefly existed in the cell cytoplasm and/or membrane of tumor cells, which was further confirmed in our study 17. Elevated level of PD-L1 expression was observed in different types of cancer. Weber et al. demonstrated that oral squamous cell carcinoma (OSCC) had higher PD-L1 expression than oral mucosa controls and elevated expression of PD-L1 was related to tumor grade and lymph node metastasis 18. Also, PD-L1 was proved to be positively related to malignancy grades and lymph node status as well as shorter patient survival in NSCLC 17. Besides, PD-L1 expression was evidently associated with age, high tumor grade of soft tissue sarcoma and had a relationship with FOXP3+ Treg infiltration 19. Moreover, Bi et al. reported that PD-L1 expression was positively related to latent membrane protein 1 (LMP1) expression in natural killer/T-cell lymphoma (NKTCL), which was probably mediated by the MAPK/NF-κB pathway and correlated with poor prognosis in NKTCL 20. Intriguingly, Dong et al. observed that AKT/mTOR pathway was activated in diffuse large B-cell lymphoma (DLBCL) cells after activating PD-1/PD-L1 signaling pathway 21. In this work, results showed that PD-L1 protein detected by IHC was higher in NSCLC tissues compared with non-cancerous control tissues, which was in line with previous studies. Meanwhile, we also demonstrated that lung SCC male patients with LNM were characterized by high expression levels of PD-L1, which needed to be further investigated and might provide predictors for PD-L1 expression. PD-L1 and HIF-1α varied in different histological types and gender, and the potential mechanisms were complicated. Among them, tobacco smoking was one of the most important differences between male and female patients, which was one of the major risks of lung squamous cell carcinoma in male patients. Chemical of cigarettes could interact with DNA and cause distinct genetic changes 22 and therefore may regulate the expression of PD-L1 and HIF-1α, so expression of PD-L1 was higher in male lung SCC patients. More potential mechanisms should be explored in the future research. In addition, our result indicated that PD-L1 was a worse prognostic factor for NSCLC patient, further providing a theoretical basis for the application of PD-1/PD-L1 blockade in immunotherapy of NSCLC.

Hypoxia is a common feature of varieties of solid cancers 23. HIF-1α was a effective transcription factor, regulating the apoptosis, proliferation and metastasis of cancer cells 24 and had a positive correlation with many genes in NSCLC, such as EGFR, p53 and so on 25. It was reported that expression of HIF-1 and HIF-2 had to do with poor prognosis, metastasis and high-grade in breast cancers 26. What's more, growing evidences suggested that HIF-1α overexpression was related to differentiation, metastasis and microvessel density of bone tumor 27. Besides, HIF-1 could regulate CD47 expression to promote evasion of phagocytosis and maintenance of cancer stem cells 28. In our study, data showed that HIF-1α was highly expressed in NSCLC tissues, but not related to clinicopathological features, which needed to be further validated in a large number of samples. Moreover, HIF-1α was proved to be a worse prognostic factor for NSCLC patients in multivariate analysis, which was consistent with above findings and provided a target for precision treatment of NSCLC.

Recent researches have provided new clues for the regulation mechanisms of PD-L1, including epigenetic, genetic, transcriptional levels and so on 29. For instance, CD274 gene amplification was a key factor driving the expression of PD-L1 and truncation of 3′UTR was related to aberrant PD-L1 expression in multifarious cancers 29, 30. Also, P53 has been implicated in regulating PD-L1 through miR-34 31 and PTEN could repress transcription and expression of PD-L1 32. Notably, binding of HIF-1α to PD-L1 promoter could stimulate the transcription of PD-L1, thereby increasing the expression of PD-L1 protein 33. In this work, PD-L1 protein was positively correlated with HIF-1α protein in NSCLC tissues. As far as we know, it was the first time to explore the relationship between PD-L1 and HIF-1α in NSCLC tissues and thereby further emphasized the role of HIF-1α in PD-L1 regulation. Meanwhile, HIF-1α was reported to enhance TAM (tumor-associated macrophages)-mediated T cell function suppression and promote tumor progression 34. HIF-1α has also been revealed to up-regulate other co-stimulatory receptors which were potential targets for immunotherapy: OX40, 4-1BB and GITR 35. In addition, HIF-1α upregulated the expression PD-L1 on MDSCs (myeloid-derived suppressor cells) and mediated the suppressive action of MDSCs and under hypoxia, the blockade of PD-L1 could enhance MDSC-mediated T cell activation and down-regulate IL-6 and IL-10 of MDSCs 36. Taken together, it was suggested that the combined targeting PD-L1 and HIF-1α may be a rationalized strategy and boost the immunotherapy for NSCLC patients.

Strikingly, our results showed that PD-L1 was a worse prognostic factor in lung ADC, while HIF-1α has no prognostic value. On contrary, HIF-1α was identified as an independent worse prognostic factor in lung SCC, but not PD-L1, which needed to be verified in further studies. It was suggested that the role of PD-L1 and HIF-1α varied in different histological types and the combined targeting PD-L1 and HIF-1α may be more important in patients with lung SCC. More clinical trials were needed to confirm this hypothesis. In conclusion, our work strongly suggested that the expression of PD-L1 and HIF-1α protein may serve as attractive worse prognostic biomarkers for NSCLC patients and the combined evaluation of PD-L1 and HIF-1α may also be valuable for prognosis. In addition, our result showed that PD-L1 protein was positively correlated with HIF-1α, which may provide evidences for a novel combinational therapy targeting PD-L1 and HIF-1α in the NSCLC patients.

## Supplementary Material

Supplementary table S1.

## Figures and Tables

**Table 1:** Association between HIF-1α and PD-L1 protein and clinicopathological features of NSCLC patients (n= 256)

Clinicopathological features	PD-L1			HIF-1α			PD-L1 and HIF-1α		
	Positive (%)	Negative (%)	*p*	Positive (%)	Negative (%)	*p*	Co-expression (%)	others (%)	*p*
**Age (years)**									
< 56 (n=110)	28 (25.5%)	82 (74.5%)	0.728	80 (72.7%)	30 (27.3%)	0.728	25 (22.7%)	85 (77.3%)	0.916
≥ 56 (n=146)	40 (27.4%)	106 (72.6%)		109 (74.7%)	37 (25.3%)		34 (23.3%)	112 (76.7%)	
**Gender**									
Female (n=65)	10 (15.4%)	55 (84.6%)	0.018*	46 (70.8%)	19 (29.2%)	0.516	9 (13.8%)	56 (86.2%)	0.041*
Male (n=191)	58 (30.4%)	133 (69.6%)		143 (74.9%)	48 (25.1%)		50 (26.2%)	141 (73.8%)	
**Histological type**									
ADC (n=133)	21 (15.8%)	112 (84.2%)	0.000*	96 (72.2%)	37 (27.8%)	0.533	18 (13.5%)	115 (86.5%)	0.000*
SCC (n=123)	47 (38.2%)	76 (61.8%)		93 (75.6%)	30 (24.4%)		41 (33.3%)	82 (66.7%)	
**Pathological grade**									
Well/moderated (n=125)	30 (24.0%)	95 (76.0%)	0.365	92 (73.6%)	33 (26.4%)	0.935	26 (20.8%)	99 (79.2%)	0.404
Poor (n=131)	38 (29.0%)	93 (71.0%)		97 (74.0%)	34 (26.0%)		33 (25.2%)	98 (74.8%)	
**Clinical stage**									
Stage I and II (n=141)	33 (23.4%)	108 (76.6%)	0.205	103 (73.0%)	38 (27.0%)	0.754	29 (20.6%)	112 (79.4%)	0.297
Stage III (n=115)	35 (30.4%)	80 (69.6%)		86 (74.8%)	29 (25.2%)		30 (26.1%)	85 (73.9%)	
**LNM status**									
LNM (n=140)	45 (32.1%)	95 (67.9%)	0.026*	106 (75.7%)	34 (24.3%)	0.451	40 (28.6%)	100 (71.4%)	0.021*
No LNM (n=116)	23 (19.8%)	93 (80.2%)		83 (71.6%)	33 (28.4%)		19 (16.4%)	97 (83.6%)	

Abbreviations: ADC: adenocarcinoma; SCC: squamous cell carcinoma; LNM, lymph node metastasis. The average age of all patients with NSCLC was 56.0±8.77 years. *: *p*<0.05.

**Table 2:** The pairwise correlation between PD-L1 and HIF-1α protein in Lung SCC and ADC

	ADC		SCC	
	PD-L1	HIF-1α	PD-L1	HIF-1α
PD-L1				
Spearman's correlation coefficient	1	0.131	1	0.218
Sig. (2-tailed)		0.134		0.003*

Values are Spearman's correlation coefficient. *P < 0.05.

**Table 3:** Summary of univariate and multivariate analysis for OS in NSCLC patients

Variables	Univariate analysis			Multivariate analysis		
	Average survival time (SE)	95% CI	*P*	Exp (B)	95.0% CI	*P*
**PD-L1**						
Positive expression	42.099 (3.999)	(34.261, 49.937)	0.006*	0.536	(0.312, 0.920)	0.024*
Negative expression	59.312 (3.121)	(53.015, 65.608)				
**HIF-1α**						
Positive expression	50.905 (3.152)	(44.727, 57.082)	0.004*	0.461	(0.256, 0.829)	0.010*
Negative expression	67.881 (5.187)	(57.713, 78.048)				
**Clinical stage**						
Stage I and II	69.316 (3.813)	(61.842, 76.790)	0.000*	0.474	(0.290, 0.775)	0.003*
Stage III	42.708 (3.255)	(36.328, 49.089)				
**LNM status**						
LNM	45.932 (2.999)	(40.053, 51.811)	0.001*	1.671	(0.994, 2.809)	0.053
No LNM	66.376 (4.007)	(58.521, 74.230)				
**Pathological grade**						
Well and moderated	62.727 (4.025)	(54.837, 70.617)	0.006*	0.580	(0.368, 0.913)	0.019*
Poor	48.993 (3.876)	(41.396, 56.591)				
**Histological type**						
ADC	54.814 (3.679)	(47.603, 62.025)	0.745	1.495	(0.906, 2.466)	0.116
SCC	58.022 (4.499)	(49.203, 66.841)				
**Gender**						
Female	61.846 (4.896)	(52.249, 71.442)	0.214	0.687	(0.403, 1.168)	0.166
Male	53.187 (3.341)	(46.638, 59.736)				
**Age**						
< 56	54.213 (3.526)	(47.301, 61.124)	0.834	0.853	(0.540, 1.345)	0.493
≥ 56	60.314 (3.761)	(52.943, 67.685)				

Abbreviations: CI: confidence interval; SE:standard error; Exp(β): odds ratio; *: *p*<0.05.
