# Supplementary material for: Co-expression of PD-L1 and HIF-1α predicts poor prognosis in Patients with Non-small Cell Lung Cancer after surgery
Source: J Cancer. 2021 Feb 2;12(7):2065–72. doi: 10.7150/jca.53119 (PMC7974520; doi:10.7150/jca.53119)
Supplement: Supplementary file 1 — Supplementary table S1. [file jcav12p2065s1.pdf]

Table S1. Summary of multivariate analysis for overall survival in lung ADC and SCC patients

Variables	ADC			SCC		
	Exp (B)	95% CI	*P*	Exp (B)	95.0% CI	*P*
**PD-L1**	0.216	(0.096, 0.484)	0.000*	0.847	(0.417, 1.722)	0.646
**HIF-α**	0.490	(0.233, 1.030)	0.060	0.321	(0.115, 0.890)	0.029*
**Clinical stage**	0.378	(0.188, 0.761)	0.006*	0.533	(0.257, 1.102)	0.090
**LNM status**	1.453	(0.723, 2.918)	0.294	2.207	(1.013, 4.807)	0.046*
**Pathological grade**	0.812	(0.447, 1.475)	0.493	0.330	(0.155, 0.705)	0.004*
**Gender**	1.100	(0.597, 2.024)	0.760	0.000	-	0.979
**Age**	0.838	(0.458, 1.531)	0.565	0.916	(0.437, 1.917)	0.815

Abbreviations: ADC: adenocarcinoma; SCC: squamous cell carcinoma; LNM: lymph node metastasis; CI: confidence interval; Exp(β): odds ratio; -: not applicable; *: $p<0.05$.
